# It Should Be Known

**Published:** 1879-08

**Authors:** 


### HALL'S JOURNAL OF HEALTH.

MONTHLY.

E. H. GIBBS, A.M., M.D., Editor,

New York.

### *IT SHOULD BE KNOWN*

That nearly all diseases are either preventable or curable; that in nine cases out of ten, when death comes before old age, it comes from culpable neglect of the health, or from improper medical treatment, and that it is blasphemy to attribute untimely deaths by disease to the dispensations of Providence.

We do not expect to make our readers entirely independent of physicians, for there are emergencies that we cannot provide against; but we propose to furnish them, each month, with the "*ounce of prevention*" against the diseases usually prevalent during that month, which will be of more value than the "*pound of cure*" should sickness come. We endeavor to teach people so to live that they may escape the ills of later years.

The Journal of Health has taken a new departure which seems to be justified by its age and its great popularity. It has heretofore confined itself to giving advice for the prevention of disease. Its field of usefulness was, therefore, circumscribed. In spite of all rules for the preservation of health, people will sometimes become ill, and it is important to know what are the best and promptest means for recovery.

During the past twenty-five years our motto has been "*When sickness comes send for a physician*" If the physician is competent—if he is accessible—and you *really* will send for him when *seriously* ill, it is by all means the best thing to do. But in most cases sickness can be prevented by an observance of the simple rules to be found in any copy of the Journal of Health. Moreover, this number, and subsequent numbers, will advise as to the best and safest treatment in all ordinary cases, directing what remedies to use and how to use them. Thousands of constitutions have been weakened, thousands of lives shortened, by the unwise use of strong drugs. We know that many diseases are cured by *perfect nursing*, *perfect rest*, *perfect foods*, *and simple and safe remedies.*

There are a few standard remedies, particularly those of Drs. Hall and Nelaton, which we have employed for years, and shall unhesitatingly recommend in all cases where they are applicable.

As for foods, which are of themselves among the best remedies, we invite attention to those prepared by the "Health Food Company" of New York—the only really scientific authority in America—to a description of whose products we devote much space in this number.

THE SUBSCRIPTION PRICE OF "HALL'S JOURNAL OF HEALTH" IS

$1.50 a year; $1 for eight months; 15 cents for a single copy; $5 for four copies one year; $10 for ten copies one year.

We will club with any other publication at $1 a year. We will send any other publication, with Hall's Journal of Health, for one dollar in addition to the price of said publication.

FOR EXAMPLE:

| | | | | | |
|---|---|---|---|---|---|
| "Scribner's Magazine" | is $4 a y'r; | "Hall's Journal of Health," | $1.50. | Both | $5 a y'r. |
| "St Nicholas'" | " 3 " | " | " | " | " 4 " |
| "Science Monthly" | " 5 " | " | " | " | " 6 " |
| "Home Journal" | " 2 " | " | " | " | " 3 " |

Agents can retain fifty cents from the subscription price or commission. No better terms will be made with any one, and all contracts to supply the Journal of Health at a lower rate are hereby revoked. It is best to send all sums less than two or three dollars in letters, sending fractions of a dollar in postage stamps. It saves trouble and expense. Most of our subscriptions come in that shape, and not a dollar has been lost in five years. Our own limit in sending money through the mails is $5. We have never lost a dollar.

We receive orders for Books, Medicines, Surgical Instruments, etc., etc.

Address E. H. Gibbs & Co.,
Publishers of Hall's Journal of Health, New York.

